# *Artemisia fragrans* Willd. Essential Oil: Chemical Profile and Insecticidal Potential against the Confused Flour Beetle, *Tribolium confusum* du Val

**DOI:** 10.3390/plants13131725

**Published:** 2024-06-21

**Authors:** Asgar Ebadollahi, William N. Setzer, Franco Palla

**Affiliations:** 1 Department of Plant Sciences, Moghan College of Agriculture and Natural Resources, University of Mohaghegh Ardabili, Ardabil 5697194781, Iran; 2 Department of Chemistry, University of Alabama in Huntsville, Huntsville, AL 35899, USA; wsetzer@chemistry.uah.edu; 3 Department of Biological, Chemical and Pharmaceutical Sciences and Technologies, University of Palermo, 90123 Palermo, Italy

**Keywords:** chemical composition, essential oil, feeding deterrence, fumigant toxicity, wormwood

## Abstract

The confused flour beetle, *Tribolium confusum* du Val, is one of the cosmopolitan and polyphagous storage insect pests. The frequent application of chemical insecticides has resulted in several side effects, including threats to human health and non-target organisms and the resistance of insect pests. In the current study, the fumigant toxicity and feeding deterrence potential of *Artemisia fragrans* Willd. essential oil on *T*. *confusum* adults were investigated. The essential oil was rich in terpenic compounds, in which α-thujone (27.8%) and 1,8-cineole (22.8%) were dominant. The essential oil displayed significant fumigant toxicity on *T. confusum*, where a concentration of 35.3 μL/L caused 100% mortality of the treated adults after 48 h. The LC_30_ and LC_40_ values (lethal concentrations to kill 30% and 40% of tested insects: 15.1 and 18.4 μL/L, respectively) significantly decreased the nutritional indices of the pest, including the consumption index, relative consumption rate, and relative growth rate. The feeding deterrence index of the essential oil were calculated as being 62.29 and 48.66% for the concentrations of 15.1 and 18.4 μL/L after 5 days, respectively. Accordingly, *A. fragrans* essential oil can be considered an efficient, available, and natural alternative to detrimental chemical pesticides in the management of *T. confusum*.

## 1. Introduction

The confused flour beetle, *Tribolium confusum* du Val (Coleoptera: Tenebrionidae), is a cosmopolitan and polyphagous insect pest of stored products, including cereal, legumes, flour, pasta, chocolate, dried fruits, and animal collections [1]. Along with direct damage caused by feeding, contamination of stored products with feces and shells of different stages of the pest leads to significant indirect damage [1]. Although the utilization of chemical insecticides is the main method used to manage such an insect pest, their frequent application results in numerous side effects, such as environmental pollution, threats to human health and non-target organisms, and the development of pest resistance [2,3]. Therefore, it is necessary to introduce efficient and low-risk agents in the management of insect pests.

The search for green strategies as a valid alternative to synthetic chemical pesticides has been active for many decades in the agricultural, pharmaceutical, food, and cosmetic industries [4,5,6], as well as in the conservation of cultural and landscape heritage [7,8,9]. Plant-derived essential oils are a complex mixture of active and volatile compounds, which are usually seen as aromatic and aliphatic compounds [10]. Terpenes, such as monoterpenes, sesquiterpenes, and diterpenes, are the main group of essential oil composition. In other words, monoterpenes and monoterpenoids (oxygenated monoterpenes), such as α-pinene and 1,8-cineole, are the dominant components of most essential oils [11]. Previous studies revealed that the essential oils isolated from several aromatic plants can be used as bio-pesticides against different groups of insect pests [12,13,14]. According to the findings of recent studies, *T. confusum* is sensitive to plant-derived essential oils. For example, the toxicity of *Artemisia dracunculus* L., *Ocimum basilicum* L., and *Rosmarinus officinalis* L. essential oils against the adults of *T. confusum* was demonstrated [15], in which the exposure of insects to a concentration of 153.8 μL/L of essential oils resulted in 93.3%, 98.3%, and 98.3% mortalities after 96 h, respectively. The adults of *T. confusum* were also susceptible to the essential oils of *Ricinus communis* L., *Eucalyptus globulus* Labill, and *Eruca sativa* Mill., and the 24 h LC_50_ values (lethal concentration to kill 50% of tested insects after 24 h) of the essential oils were 25.3, 33.5, and 38.0 mg/L, respectively [16]. The fumigant toxicity of the essential oil of *Myrtus communis* L. with a 24 h LC_50_ value of 247.0 μL/L was also reported against the adults of *T. confusum* [17]. Along with lethal effects, the essential oils can show sub-lethal activities against insect pests. For instance, the essential oil of *R. officinalis* displayed lethal fumigant toxicity and sub-lethal antifeedant effects against the adults of *T. confusum* [18].

There are about 500 aromatic species in the *Artemisia* L. genus, which is the most prominent member of the Asteraceae family, known as wormwood, and widely distributed throughout the world [19]. *Artemisia fragrans* Willd. has aromatic leaves and flowers and is about 45 cm tall. The leaves of this aromatic plant tend to be white at first due to the presence of numerous trichomes, which are lost as the plant continues to grow [20]. *A. fragrans* has spread widely in Iran and is one of the dominant plants in the north of Ardabil province, especially in the rangelands of the Mughan region [21]. Although aliphatic compounds can be detected, the essential oil of *A. fragrans* contains various terpenic compounds from hydrocarbon monoterpenes and monoterpenoids to sesquiterpenes [22]. The terpenes 1,8-cineole, α-thujone, α-pinene, β-pinene, camphor, and camphene are among the dominant compounds in *A. fragrans* essential oil [23,24,25]. Different biological effects of the essential oil of *A. fragrans*, including antioxidant, antibacterial, antimalarial, antileishmanial, and even herbicidal activities, were reported in previous studies [25,26,27,28].

Although the possibility of insect pest management by essential oils extracted from several species of *Artemisia* genus has been reported in recent studies [29,30,31], the insecticidal potential of *A. fragrans* essential oil has not been investigated yet. Accordingly, the fumigant toxicity of the essential oil extracted from the aerial parts of *A. fragrans* as an available natural agent was studied against *T. confusum*. In addition to the acute toxicity, the nutritional indices of the pest treated with essential oil were also investigated. The chemical composition of the essential oil was analyzed, and the relationship between the identified components and the pesticidal properties of the essential oil was discussed.

## 2. Results

### 2.1. Chemical Analysis of Essential Oil

The *A. fragrans* essential oil was analyzed by GC-MS (gas chromatography–mass spectrometry). A total of 54 compounds were identified in the essential oil, which accounted for 92.7% of the total composition (Table 1). The essential oil was dominated by two oxygenated monoterpenoids, 1,8-cineole (22.8%) and α-thujone (27.8%). The yield of essential oil extraction was 1.23 ± 0.14 (w/w).

### 2.2. Fumigant Toxicity of Essential Oil

**Figure 1:**
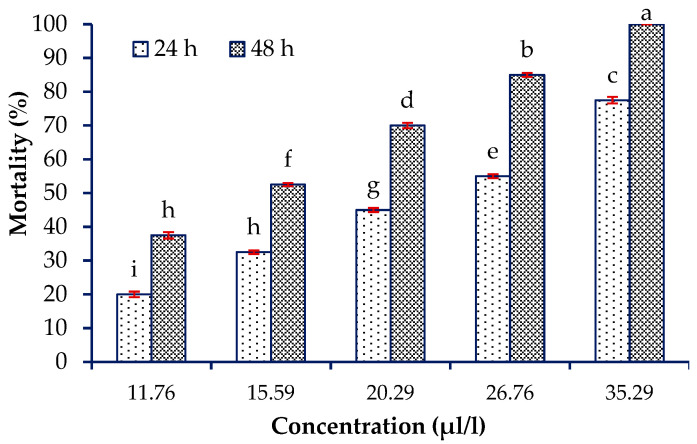
Mean mortality percentage (±SE) of the *Tribolium confusum* du Val exposed to different concentrations of *Artemisia fragrans* Willd. essential oil after 24 and 48 h. Different letters display significant differences between the corresponding means.

The data obtained from the fumigant toxicity of the *A. fragrans* essential oil on the adults of *T. confusum* had a normal distribution according to the Kolmogorov–Smirnov test (Z = 0.56 and sig. (two-tailed) = 0.91). There was no mortality in the control group after 24 and 48 h exposure times. The effects of different concentrations of the *A. fragrans* essential oil (F = 93.62; df = 4, 30; *p* < 0.001) and 24 and 48 h exposure times (F = 113.36; df = 1, 30; *p* < 0.001) were significant to the mortality of the insect pests. However, the interaction effect of the essential oil concentration and exposure time to the pest was not significant (F = 0.99; df = 4, 30; *p* = 0.427). The *Artemisia fragrans* essential oil had a high fumigant toxicity on the *T. confusum*, where the concentration of 35.3 µL/L from the essential oil caused 100% mortality of the treated adults after 48 h (Figure 1).

According to the results of the Probit analysis shown in Table 2, the LC_50_ value of the *A. fragrans* essential oil was decreased from 22.13 μL/L at 24 h of exposure time to 14.70 μL/L after 48 h. The LC_30_ and LC_40_ values were used to evaluate the antinutritional effects of the essential oil. The values of the correlation coefficients (0.98 and 0.99 for 24 and 48 h exposure times) indicate a positive and direct relationship between the pest mortality and the essential oil concentrations.

### 2.3. Antifeedant Effects of Essential Oil

**Figure 2:**
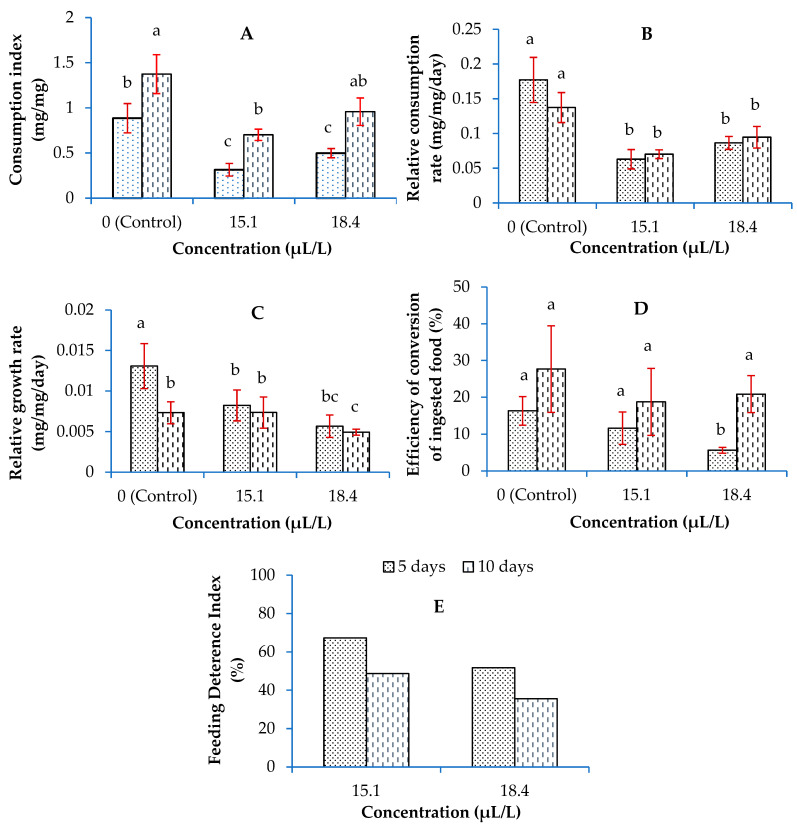
Effects of the LC_30_ and LC_40_ values of the *Artemisia fragrans* Willd. essential oil on the nutritional indices, including the consumption index (**A**), relative consumption rate (**B**), relative growth rate (**C**), efficiency of conversion of ingested food (**D**), and feeding deterrence index (**E**) of *Tribolium confusum* du Val after 5 and 10 days of exposure time. Different letters designate significant differences between the corresponding means according to Tukey’s test at the probability level of 5%.

The effect of the 24 h LC_30_ and LC_40_ values of the *A. fragrans* essential oil (15.1 and 18.4 μL/L, respectively) on the nutritional indices of the *T. confusum* adults, including the consumption index, relative consumption rate, relative growth rate, and efficiency of conversion of ingested food after 5 and 10 days, are shown in Figure 2. The consumption index was significantly affected by the essential oil concentrations (F = 9.93; df = 2, 24; *p* < 0.05) and exposure times (F = 14.82; df = 1, 24; *p* < 0.05), where it was significantly decreased for the insects treated with 15.1 and 18.4 μL/L of essential oil after 5 days compared with the control group (Figure 2A).

The relative consumption rate of *T. confusum* adults was also decreased by treating with LC_30_ and LC_40_ values of the *A. fragrans* essential oil. Even though the increase in time did not affect the relative consumption rate (F = 0.26; df = 1, 24; *p* = 0.62), the value of this index was significantly reduced by the tested concentrations at both 5 and 10 days of exposure time (F = 11.39; df = 2, 24; *p* < 0.05) (Figure 2B).

The use of the studied concentrations of *A. fragrans* essential oil reduced the relative growth rate of the pest compared with the control group (F = 3.43; df = 2, 24; *p* = 0.04); the lowest value was seen for the adults treated by the highest tested concentration. However, it was not affected by the exposure time (F = 2.56; df = 1, 24; *p* = 0.12) (Figure 2C). The effect of the essential oil concentrations (F = 0.08; df = 2, 24; *p* = 0.46) and exposure time of insect pest (F = 3.60; df = 1, 24; *p* = 0.07) on the efficiency of converting the eaten food were not significant (Figure 2D).

The feeding deterrence index of the *A. fragrans* essential oil at a concentration of 15.1 μL/L was calculated as being 62.29 and 51.75% after 5 and 10 days, respectively. The corresponding values with the concentration of 18.4 µL/L were 48.66 and 35.58%, respectively (Figure 2E).

## 3. Discussion

The insecticidal effects of essential oils isolated from different *Artemisia* species were demonstrated in previous studies. For example, the *Artemisia annua* L. essential oil showed significant fumigant toxicity, with a 24 h LC_50_ of 3.34 μL/L against the fourth-instar larvae of the mulberry pyralid *Glyphodes pyloalis* Walker [31]. The essential oils of *Artemisia absinthium* L. and *Artemisia dracunculus* L. displayed noticeably fumigant toxicity, with LC_50_ values of 2.60 and 1.08 µL/L, respectively, against eggs of the potato tuber moth *Phthorimaea operculella* (Zeller) [32]. Toxicity of essential oils isolated from four *Artemisia* species, including *A. dalai-lamae* Krasch., *A. tangutica* Pampanini, *A. tanacetifolia* L., and *A. ordosica* Krasch., where 24 LC_50_ values of 25.7, 17.4, 41.9, and 21.7 µg/insect, respectively, against the red flour beetle *Tribolium castaneum* Herbst were documented [33]. However, in the present study, the insecticidal effects of *A. fragrans* essential oil was evidenced for the first time, in which the essential oil with a 24 h LC_50_ of 22.13 μL/L had noteworthy fumigant toxicity against the adults of *T. confusum*. The different lethal concentrations in the abovementioned and present studies may be justified by differences in the tested insect pests and *Artemisia* species.

Along with acute toxicity, based on the results of the present study, the *A. fragrans* essential oil had anti-nutritional effects on the adults of *T. confusum*: A significant reduction in consumption index, relative consumption rate, and relative growth rate, and a 62.29% feeding deterrence index at a concentration of 15.1 μL/L after 5 days. There was also a decrease in the consumption index and relative consumption rate of the pest treated with *A. fragrans* essential oil actually shows that the insect refused to eat food. The inhibitory compounds in the essential oil probably interfered with the functioning of the pest’s feeding stimulation signals. It may also be said that given the reduction in the relative growth rate of insects, which can be related to a decrease in the relative consumption rate and conversion efficiency of the digested food, the utilization quantity of food also decreased [34]. In general, decreasing nutritional indices result in low biological performance of the pest, especially in terms of food absorption and growth. In consonance with the results of the present study, nutritional indices falling in the insect pests treated by *Artemisia* essential oils was documented in recent years: The relative growth rate and efficiency of conversion of ingested food of Egyptian cotton leaf worm *Spodoptera littoralis* (Boisduval) larvae treated with essential oil of *Artemisia monosperma* Del. decreased [35]. In the other study, a 53.4% feeding deterrence index was displayed for the *S. littoralis* larvae treated by 100 μg/cm^2^ of *A. dracunculus* essential oil [36].

**Figure 3:**
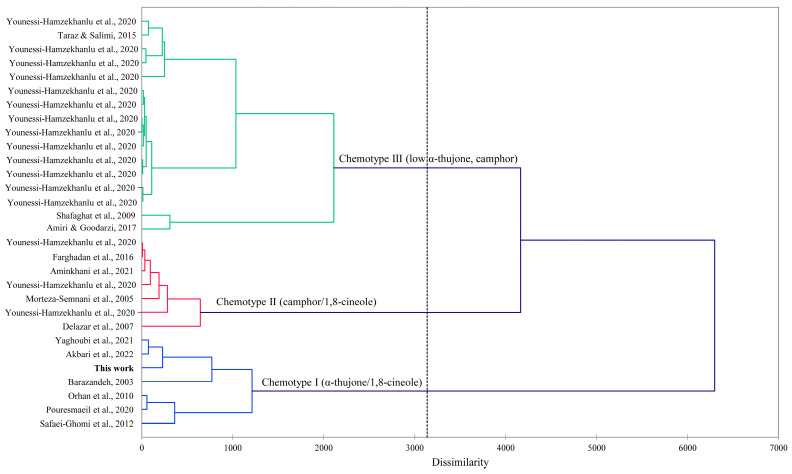
Dendrogram based on hierarchical cluster analysis (HCA) of *Artemisia fragrans* Willd. essential oil compositions. Akbari et al. 2022 [25], Aminkhani et al. 2021 [24], Amiri and Goodarzi 2017 [40], Barazandeh 2003 [41], Delazar et al. 2007 [42], Orhan et al. 2010 [26], Farghadan et al. 2016 [43], Morteza-Semnani et al. 2005 [37], Pouresmaeil et al. 2020 [27], Safaei-Ghomi et al. 2012 [44], Shafaghat et al. 2009 [45], Taraz and Salimi 2015 [46], Yaghoubi et al. 2021 [47], and Younessi-Hamzekhanlu et al. 2020 [23].

The terpenes comprising α-thujone (27.8%), 1,8-cineole (22.8%), *p*-cymen-7-ol (3.5%), terpinen-4-ol (2.9%), β-thujone (2.8%), borneol (2.7%), *trans*-*p*-menth-2-en-1-ol (2.3%), and α-pinene (2.0%) were identified as the main compounds in the *A. fragrans* essential oil in the present study. The chemical profile of the *A*. *fragrans* essential oil was explored in previous studies. Morteza-Semnani et al. [37] revealed that the terpenes camphor (46.0%), 1,8-cineole (23.7%), camphene (7.9%), borneol (4.9%), and chrysanthenone (3.4%) were the prominent compounds in the essential oil isolated from the flowering aerial parts of *A*. *fragrans*. In the present study, camphor (1.1%), camphene (1.9%), and borneol (2.7%) with different quantities were also identified, but no trace of chrysanthenone was seen. It was also found that the chemical profile of the *A*. *fragrans* essential oil can be varied based on the chemotypes and different parts of the plant. For example, according to the results of Saedi et al. [38], there were two different chemotypes among the essential oils of populations of *A. fragrans* in East Azerbaijan province, Iran. Artemisyl acetate (20.9%), 1,8-cineole (17.2%), borneol (8.0%), β-ocimene (7.8%), camphor (7.2%), bornyl acetate (4.1%), and camphene (2.5%) were prominent in the first chemotype. In the second chemotype, the chemical profile was significantly different, in which chrysanthenone (47.5%) had the highest amount, followed by 1,8-cineole (6.0%), β-thujone (5.2%), caryophyllene oxide (4.1%), and pinocarvone (3.2%). According to the study of Aminkhani et al. [24], camphor (33.9%), 1,8-cineole (23.5%), terpinene-4-ol (3.1%), artemisyl acetate (3.0%), and camphene (2.9%) in the essential oil of leaves, and camphor (27.3%), 1,8-cineole (27.0%), terpinene-4-ol (4.0%), borneol (3.1%), and carvacrol (3.0%) in the essential oil isolated from flowers of *A*. *fragrans* were the main compounds. In another investigation, the chemical composition of the *A*. *fragrans* essential oil from three provinces of Ardabil, East Azerbaijan, and West Azerbaijan of Iran were investigated. The results showed that camphor (9.9–34.4%), α-thujone (19.2–42.6%), and 1,8-cineole (12.6–31.9%) were the dominant compounds [23]. Three chemotypes based on the chemical compositions were identified in this study: chemotype I, α-thujone/1,8-cineole; chemotype II, camphor/1,8-cineole; and chemotype III, which showed relatively low concentrations of α-thujone or camphor. In order to place the essential oil composition of *A. fragrans* in this work with previously reported compositions, a hierarchical cluster analysis was carried out based on the major essential oil components (Figure 3). The cluster analysis was in excellent agreement with Younessi-Hamzekhanlu et al. [23], and firmly placed the essential oil in this current work into the α-thujone/1,8-cineole chemotype. In general, the chemical composition of plant essential oils may be changed by various exogenous and endogenous factors, such as climate conditions, geographical location, plant growth stage and ecotype, and the method of essential oil extraction [39]. Therefore, the differences observed in the identified compounds of *A*. *fragrans* essential oil in the present study and the abovementioned studies could have originated due to such factors.

Recent research results show that the insecticidal properties of *Artemisia* essential oils are directly related to their chemical compositions [30,36,48]. Additionally, several reports are on the insecticidal potential of the identified compounds in the *A*. *fragrans* essential oil. For example, based on the toxicity against the larvae, pupae, and adult stages, Kheloul et al. [17] stated that 1,8-cineole or essential oils containing large amounts of this monoterpenoid may have high insecticidal potential against *T. confusum*. In another study, the fumigant toxicity of 1,8-cineole, as one of the main compounds of *Artemisia nakaii* Pamp., with a 24 h LC_50_ value of 7.00 μL/L determined for third-instar larvae of *Spodoptera litura* Fab., gave *A. nakaii* essential oil insecticidal properties [30]. Xie et al. [49] indicated that among the main compounds of *Seriphidium brevifolium* (Wall. ex DC.) essential oil, (α + β) thujone and 1,8-cineole had considerable fumigant toxicity against workers of the red imported fire ant *Solenops isinvicta* Buren, with LC_50_ values of 17.7 and 30.7 μL/L after 12 h of exposure. Different insecticidal modes of action were also reported in this research: inhibitory effects on acetylcholinesterase and carboxylesterase activity by 1,8-cineole and (α + β) thujone, respectively. They concluded that *S. brevifolium* essential oil and the monoterpenes (α + β) thujone and 1,8-cineole could be developed as eco-friendly agents for managing red imported fire ants. The insecticidal potential of borneol, bornyl acetate, camphene, camphor, carvacrol, *p*-cymene, spathulenol, terpinene-4-ol, and α-pinene, as well as some other compounds recognized in the *A*. *fragrans* essential oil, were also documented [50,51,52,53]. It can be concluded that the observed insecticidal effects of *A*. *fragrans* essential oil may be due to the presence of such pesticidal compounds and their interactions.

The results of the present study showed that the fumigation of the *A. fragrans* essential oil could cause high mortality in the adults of *T*. *confusum*. Moreover, if the pest was affected by the LC_30_ and LC_40_ values, its nutritional indices, including the consumption index, relative consumption rate, and relative growth rate, diminished. The reduction in these indices may result in critical disruption in the biological processes of the pest, especially in food absorption and growth. Recent studies showed that *Artemisia* essential oils displayed other modes of action, such as the inhibition of digestive (α-amylases, proteases, lipases, and α- and β-glucosidases) and detoxifying enzymes (acetylcholinesterase and glutathione-s-transferase) activity; reductions in protein, glucose, triglyceride amounts, and hemocyte numbers; the deterioration of digestive cells of the larval midgut; and a decrease in yolk spheres in the ovaries of emerging adults of the insect pests [31,35,54]. Indeed, according to recent studies [55,56], insect pests can detoxify essential oils by increasing the activities of detoxifying enzymes, such as esterases and glutathione-S-transferase. However, due to the multiple modes of action of essential oils and their compounds, insect pests are susceptible and their chance of developing resistance against these natural agents will be low. In contrast, the possibility of pest resistance to chemical pesticides has been high due to simple modes of action.

## 4. Materials and Methods

### 4.1. Plant Materials and Essential Oil Extraction

Aerial parts of *Artemisia fragrans* were collected from the pastures of the Khoroslu region, Ardabil province, Iran (39°15′21.0″ N 48°01′13.0″ E), in May–June 2023. The plant species was identified according to the keys described by Asri [57]. The collected specimens were dried in the shade in the laboratory within one week and powdered using an electric grinder (IKA^®^, M20, Königswinter, Germany). Essential oil extraction was performed using a glass Clevenger apparatus with 50 g of dry plant powder and 1000 mL of distilled water within 180 min. The extracted essential oil was dehydrated with sodium sulfate and stored in glass containers with aluminum coating at 4 °C until use. The yield of essential oil extraction was calculated using the following equation [58]:

Yield (%) = weight of obtained essential oil/weight of dry plant × 100

### 4.2. Chemical Analysis of Essential Oil

The chemical composition of the *A. fragrans* essential oil was analyzed using a gas chromatograph (Agilent 7890B, Santa Clara, CA, USA) connected to a mass spectrometer (Agilent 5977A). The length, diameter, and thickness of the gas chromatography column (HP-5ms) were 30 m, 0.25 mm, and 0.25 µm, respectively. The essential oil solution was prepared by diluting in methanol (1:10). The solution (1 µL) was injected at 250 °C, and helium was used as a carrier gas at a rate of 0.1 mL per minute. Retention index (RI) values were determined using a homologous series of n-alkanes [59]. The essential oil compositions were ascertained by a comparison of their RI values and MS fragmentation patterns with those reported in the databases [60,61].

### 4.3. Insect Rearing

The initial population of *T. confusum* was gathered from infected flour in the Khoroslu region, Ardabil province, Iran. Rearing of the pest was carried out inside cylindrical containers whose opening was covered with net fabric for ventilation. The pest was reared in the laboratory for at least 3 generations. Adults (100 insects) were released into the containers on 200 g wheat flour (Zagros) to obtain synchronized insects. The containers were kept in the growth chamber with a temperature of 28 ± 2 °C, relative humidity of 65 ± 5%, and 24 h darkness. The adult insects were separated from the breeding containers, and the flour containing the pest eggs was kept. Synchronized adult insects (1–7 days old) were used for the experiments.

### 4.4. Fumigant Toxicity of Essential Oil

To evaluate the fumigation toxicity of the *A. fragrans* essential oil, ten adult insects (1–7 days old) were transferred to 340 mL glass containers with a diameter of 7.5 cm and a height of 9.2 cm as a fumigation chamber. Adult insects were treated with concentrations that were responsible for about 25–75% insect mortality, which were calculated according to the results of the primary experiment based on logarithmic distance (11.76, 15.59, 20.29, 26.76, and 35.29 µL/L). Concentrations were poured on filter paper pieces with a diameter of 3 cm. The treated filter papers were glued to the inner surface of the fumigant containers, and the screw lids of the containers were closed in an air-tight manner. Then, the containers comprising treated insects were maintained in a growth chamber at a 28 ± 2 °C temperature, 65 ± 5% relative humidity, and 24 h darkness. The experiments were repeated four times and the insect mortalities were recorded after 24 and 48 h. In the control group, all steps were repeated, except for the addition of essential oil concentrations.

### 4.5. Antifeedant Effects of Essential Oil

To assess the effects of the *A. fragrans* essential oil on the nutritional indices of *T. confusum*, two hundred adults were treated with LC_30_ (15.09 µL/L) and LC_40_ (18.40 µL/L) values (lethal concentrations to kill 40% and 50% of tested insects, respectively) of essential oil, which were calculated based on fumigant bioassays. After 24 h, the surviving insects were separated into five replicates of 10 adults separately for each concentration and the control and transferred inside 6 cm Petri dishes containing 2 g wheat flour. The weight of the insects before and after feeding, the weight of the given food, and the weight of the remaining food at the end of the experiment were documented after 5 and 10 days using a digital scale (Sartorius AG, GCA803S, Göttingen, Germany). To measure the percentage of dry weight of adult insects and flour, the studied samples were weighed, dried in an oven at 60 °C for 48 h, and then weighed again [56]. The nutritional indices, including the consumption index (CI), relative consumption rate (RCR), relative growth rate (RGR), and efficiency of conversion of ingested food (ECI) was measured using the following formulae [62]:

CI = F/A

RCR = F/TA

RGR = G/TA

ECI = G/F

where F is the dry weight of the food eaten (mg), A is the mean dry weight of the insects during the feeding period (mg), T is the feeding period (days), and G is the dry weight obtained during the feeding period (mg). Also, the feeding deterrence index (FDI) was calculated using the following formula [63]: FDI = [(C − T)/C] × 100, in which C and T are the mean weights of the food eaten in the control and treatment groups, respectively.

### 4.6. Statistical Analysis

The normality of the data related to the fumigant toxicity of *A. fragrans* essential oil was tested using the Kolmogorov–Smirnov test. The results of all experiments were analyzed by variance analysis and compared using Tukey’s test at the probability level of 5%. A probit analysis was performed to achieve lethal concentrations and associated regression lines. The statistical analyses were performed using SPSS version 16 software.

HCA (hierarchical cluster analysis) was carried out using XLSTAT v. 2018.1.1.62926 (Addinsoft, Paris, France). The concentrations of the 10 most abundant components (camphene, *p*-cymene, 1,8-cineole, filifolone, α-thujone, β-thujone, chrysanthenone, camphor, filifolide A, and (*E*)-β-caryophyllene) from this study, as well as previously reported compositions from the literature [23,24,25,26,27,37,40,41,42,43,44,45,46,47], were used for this analysis. Dissimilarity was used to determine the clusters considering the Euclidean distance, and Ward’s method was used to define agglomeration.

## 5. Conclusions

The promising fumigant toxicity and antifeedant effects of *A. fragrans* essential oil, which is rich in insecticide terpenes, such as 1,8-cineole and α-thujone, against *T. confusum* were realized in the present study. It should also be noted that the plant samples in this study were abundantly grown in the north of Ardabil province, Iran, and it is possible to obtain a large amount of it if needed. Therefore, *A. fragrans* essential oil can be introduced as an effective and available natural control agent for *T*. *confusum*. The investigation of the other insecticidal activities of *A. fragrans* essential oil, such as the effect on demographic parameters and its modes of action, can be effective in its application. Also, the preparation of new formulations of the essential oil, such as nanocapsules and nanoemulsions, with controlled-release capability in the application of current findings may be effective. Furthermore, for the development of *A. fragrans* essential oil as a natural insecticide, further research should be focused on its safety in humans.

## Figures and Tables

**Table 1:** Chemical analysis of the essential oil isolated from the aerial parts of *Artemisia fragrans* Willd.

RI_calc_	RI_db_	Compounds	%	RI_calc_	RI_db_	Compounds	%
844	847	(*Z*)-Salvene	0.3	1237	1238	Cuminal	1.2
929	932	α-Pinene	2.0	1242	1239	Carvone	0.8
945	946	Camphene	1.9	1244	1244	Carvotanacetone	0.2
969	969	Sabinene	tr	1249	1249	Piperitone	0.8
978	974	β-Pinene	0.3	1251	1255	Carvenone	0.2
985	979	Octan-3-one	0.1	1254	1253	*trans*-Sabinene-hydrate acetate	0.3
991	988	Myrcene	0.6	1283	1287	Bornyl acetate	1.0
1029	1025	*p*-Cymene	1.3	1294	1289	*p*-Cymen-7-ol	3.5
1034	1026	1,8-Cineole	22.8	1298	1299	Terpin-1-en-4-yl acetate	0.4
1053	1054	γ-Terpinene	0.4	1304	1298	Carvacrol	1.4
1100	1101	α-Thujone	27.8	1315	1316	δ-Terpinyl acetate	0.4
1118	1112	β-Thujone	2.8	1322	1324	Myrtenyl acetate	0.2
1122	1118	*cis-p*-Menth-2-en-1-ol	0.8	1345	1346	α-Terpinyl acetate	0.4
1134	1139	Camphor	1.1	1352	1356	Eugenol	0.2
1136	1136	*trans-p*-Menth-2-en-1-ol	2.3	1380	1376	Methyl (*E*)-cinnamate	0.6
1140	1140	*trans*-Verbenol	1.5	1398	1392	(*Z*)-Jasmone	0.8
1150	1154	Sabina ketone	0.4	1416	1417	(*E*)-β-Caryophyllene	0.7
1154	1160	Pinocarvone	0.7	1445	1454	Geranyl acetone	0.1
1159	1155	*iso*-Borneol	1.3	1449	1452	α-Humulene	0.1
1166	1165	Borneol	2.7	1483	na	*p* Menthane-1,2,4-triol	0.2
1178	1174	Terpinen-4-ol	2.9	1574	1574	γ-Undecalactone	0.2
1182	1179	*p*-Methylacetophenone	0.3	1583	1577	Spathulenol	1.0
1185	1183	Cryptone	0.3	1587	1582	Caryophyllene oxide	0.6
1191	1185	*p*-Cymen-8-ol	1.0	1662	1668	14-Hydroxy-9-*epi*-(*E*)-Caryophyllene	0.2
1195	1186	α-Terpineol	0.9			Monoterpene hydrocarbons	4.5
1198	1195	*cis*-Piperitol	1.0			Oxygenated monoterpenoids	83.8
1202	1194	Myrtenol	0.5			Sesquiterpene hydrocarbons	0.9
1220	1207	*trans*-Piperitol	1.3			Oxygenated sesquiterpenoids	1.6
1223	1204	Verbenone	0.5			Benzenoid aromatics	1.2
1234	1227	*p*-Cumenol	0.3			Others	1.7
						Total identified	92.7

RI_calc_—retention index calculated with respect to a homologous series of n-alkanes on an HP-5ms column. RI_db_—reference retention indices from the databases. tr—trace (<0.05%). na—reference retention index not available.

**Table 2:** Probit analysis of the mortality of *Tribolium confusum* du Val adults treated by *Artemisia fragrans* Willd. essential oil after 24 and 48 h of exposure time.

Time (h)	Lethal Concentrations with 95% Confidence Limits (μL/L)				Intercept	Slope	χ^2^ (df = 3)	Sig.*	R^2^
	LC_30_	LC_40_	LC_50_	LC_90_					
24	15.09 (13.23–16.61)	18.40 (16.69–20.02)	22.13 (20.34–24.27)	56.42 (46.14–76.97)	−4.24	3.15	1.91	0.59	0.98
48	11.30 (9.92–12.42)	12.94 (11.69–14.01)	14.70 (13.52–15.74)	27.96 (25.47–31.73)	−5.36	4.59	7.33	0.06	0.99

* Since the significance level was greater than 0.05, no heterogeneity factor was used in the calculation of the confidence limits. LC_30_, LC_40_, LC_50_, and LC_90_ values are the lethal concentrations to kill 30, 40, 50, and 90% of tested insects, respectively. Sig.: significant.

## Data Availability

The data that support the findings of this study are available upon reasonable request.
